# THE PROTECTIVE EFFECT OF RESILIENCE ON SHRINKAGE OF FRIENDSHIP IN LATER LIFE

**DOI:** 10.1093/geroni/igac059.2400

**Published:** 2022-12-20

**Authors:** Ikuko Sugawara, Midori Takayama, Yoshiko Ishioka

**Affiliations:** Bunri University of Hospitality, Suginami-ku, Tokyo, Japan; Keio University, Yokohama, Kanagawa, Japan; O.P. Jindal Global University, Sonipat, Haryana, India

## Abstract

The purpose of this study was to examine the protective effect of trait resilience, a positive personality characteristic that enhances individual adaptation to life events and adversity, in the face of declining social relationships in later life. Social relations have a significant effect on our development and well-being throughout life. However, it is known that a decline in physical, cognitive, and mental functioning makes it difficult to maintain active participation in society and leads to shrinking social networks in later life. Therefore, it is important to identify protective factors that enable people to maintain positive social relations when functioning in daily life declines in old age. We analyzed longitudinal survey data from a representative sample of older Japanese adults aged 74 to 86 years (N=1064). The interaction effect of resilience in the relationship between functions (physical, cognitive, and mental) and relationships with friends (social support exchange and companionship) was examined using multi-group Structured Equation Modeling (SEM). Respondents were divided into upper quartile and lower quartile groups according to their Resilience Scale (RS-14) scores. The results showed significant group differences for the effect of cognitive function on friendship. The positive correlation between cognitive functioning and friendship was stronger among respondents with low resilience. For high resilience participants, cognitive functioning was not associated with friendship. The results suggest that resilient older adults cope well with poor cognitive health and maintain positive social relationships in very late life.

